# TLR4 signaling cascades leading to increases in albumin permeability, nephrin ectodomain shedding, and apoptosis in MPC‐5 podocytes

**DOI:** 10.14814/phy2.71147

**Published:** 2026-10-16

**Authors:** Eun Young Kim, Stuart E. Dryer

**Affiliations:** ^1^ Department of Biology and Biochemistry University of Houston Houston Texas USA; ^2^ Department of Biomedical Sciences Tilman J. Fertitta Family College of Medicine, University of Houston Houston Texas USA

**Keywords:** effacement, pattern recognition receptor, podocyte, slit diaphragm

## Abstract

Proteinuria is a cardinal symptom of kidney disease, but common transient proteinurias may reflect a normal physiological response to distant tissue damage or infection. Here we observed that activation of Toll‐like receptor‐4 (TLR4) using the selective agonist CRX572 induced three distinct functional effects in cultured podocytes. TLR4 activation evoked a reversible increase in albumin flux (*P*_Alb_) across a confluent layer of podocytes in 60 m or less. At 24 h matrix metalloproteinase‐dependent shedding of nephrin ectodomains was detected. Finally, apoptosis occurred after 72 h. These effects were blocked by TAK242, a selective TLR4 antagonist. TLR4 activation engaged multiple intracellular transduction cascades. Activation of Erk was observed at 30 m, increased reactive oxygen species (ROS) was seen at 60 m and 24 h, and activation of NFκB was seen with 24 h of TLR activation. The increase in *P*_Alb_ required increased ROS and activation of Erk. The subsequent nephrin ectodomain shedding also required Erk signaling but did not require increased cytosolic ROS or NFκB activation. Finally, TLR4‐evoked apoptosis required both increased ROS and activation of NFκB but did not require Erk activation. Innate immune signaling through TLR4 may alter glomerular function via parallel pleiotropic signaling cascades in podocytes.

## Graphical abstract

Activation of TLR4 receptors in cultured podocytes evokes multiple functional responses which occur over different time scales, including an increase in albumin permeability (1), shedding of nephrin ectodomains (2) and apoptosis (3). Each of these responses requires a distinct set of intracellular signaling pathways.

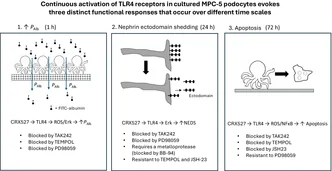

## 1 INTRODUCTION

Sustained proteinuria is a cardinal symptom of kidney disease, including chronic progressive disorders that lead to end‐stage kidney failure. Increased urine albumin excretion occurs in circulating factor diseases such as primary focal and segmental glomerulosclerosis, various forms of glomerulonephritis, certain genetic disorders, and secondary to metabolic, physiological, or infectious diseases that adversely affect renal function. However, not all instances of proteinuria reflect a significant kidney disease state. For example, transient proteinuria has long been known to occur with minor infections, especially in children (Cho & Kim, 2017; Vehaskari & Rapola, 1982). One analysis found albuminuria in 10% of a population of nearly 9000 school children, and nephrotic range urinary protein concentrations >1000 mg/dL were frequently detected (Vehaskari & Rapola, 1982). Importantly, in most cases this proteinuria resolved quickly and was not associated with any measurable decline in renal function. Transient proteinuria has not been as well documented in adults, in part because urine albumin excretion is not typically measured unless there is already a reason to suspect proteinuria, and because most adults will not seek clinical attention because of for example, a minor respiratory infection. However, it has been observed following prolonged or intense exercise and/or elevated body temperature (Bellinghieri et al., 2008; Ravanelli et al., 2021). Transient proteinuria is probably a common phenomenon even in the absence of kidney disease.

Specialized junctions formed between the foot processes of podocytes, known as slit diaphragms, form the terminal and most size‐selective component of the glomerular filtration barrier (Haraldsson et al., 2008). Podocyte stress arising from various factors results in a complex ultrastructural change known as foot process effacement, characterized by broadening and retraction of foot processes, accompanied by marked changes in cytoskeletal organization and a loss of slit diaphragms and their replacement by other types of junctions (Kriz et al., 2013; Kriz & Lemley, 2017; Pavenstädt et al., 2003). Effacement is, at least initially, a reversible cellular adaptation to prevent detachment of podocytes from the glomerular basement membrane (Kriz et al., 2013; Pavenstädt et al., 2003), which is important because there is only a limited capacity to regenerate podocytes should they detach or die.

Podocytes have additional functions. For example, they can function as antigen‐presenting cells in a manner similar to macrophages or dendritic cells (Li et al., 2020). Similar to many types of immune cells, they also express multiple classes of pattern‐recognition receptors (PRRs), including several located on the cell surface (Myette et al., 2025). PRRs are a diverse group of receptors that are activated by molecules associated with pathogens (PAMPs), or that are released from damaged, senescent, or dying cells (DAMPs). The agonists of PRRs include small metabolites, or full‐length proteins or their degradation products, many of which are potentially toxic. Activation of PRRs plays a role in initiating innate immune responses, for example by driving immune cell migration to sites of infection or tissue injury and by secretion of cytokines such as TNFα and IL‐1β.

In this study we have focused on signaling through Toll‐like receptor‐4 (TLR4), a PRR that has long been known to be expressed in podocytes (Banas et al., 2008; Reiser et al., 2004). Bacterial lipopolysaccharides (LPS), endotoxins released from the outer membranes of gram‐negative bacteria, are prototypical agonists of TLR4. Injection of LPS into mice is widely used as a model for sepsis (Dickson & Lehmann, 2019). It also results in proteinuria that can be detected 24 h later that is associated with effacement of foot processes (Lorenzen et al., 2008; Reiser et al., 2004). Importantly, the glomerular responses to a single injection of LPS are fully reversible (Reiser et al., 2004). This has led to the hypothesis that proteinuria may be a normal response in certain circumstances, as it could allow more rapid clearance of substances that might be toxic if allowed to accumulate.

Relatively little is known about TLR4 signaling in podocytes. In vivo studies of TLR4 agonists are difficult to interpret, as these receptors are widely distributed on monocytes, macrophages, dendritic, and epithelial cells, and their activation results in the release of cytokines into the circulation that can in turn impact the entire organism, including significant hemodynamic effects. Therefore, for this study we have conducted in vitro experiments on podocytes in the absence of other cell types, focusing on three distinct cellular processes of podocytes: albumin permeability (*P*_Alb_), shedding of soluble nephrin ectodomains, and apoptotic cell death. Previous studies have shown that cultured podocytes form nephrin‐dependent barriers with an ultrastructure and selectivity similar to those seen at glomerular filters in vivo (Hunt et al., 2005; Reiser et al., 2000; Shankland et al., 2007). Using a highly selective TLR4 agonist, we have found that TLR4 activation affects all three of these processes, albeit with markedly different time courses and through distinct transduction pathways. We have also examined the role of three different intracellular signaling cascades in driving these processes, including generation of reactive oxygen species (ROS), activation of Erk MAP kinase, and activation of NFκB at various time points.

## 2 MATERIALS AND METHODS

### 2.1 Cell culture

All experiments utilized an immortalized mouse podocyte cell line (MPC‐5 cells) originally developed by Dr. Peter Mundel, who provided them to us. The passage number of the cells used in these experiments was <15. Cells were propagated at 33°C at a low density and differentiation into a podocyte phenotype was induced by removal of γ‐interferon (Sigma‐Aldrich catalog number IF005) and a temperature switch to 37°C for 14 days as described previously (Kim et al., 2008). The differentiated podocytes robustly express nephrin and podocin.

### 2.2 Albumin flux assays

The methods used here were described previously (Kim & Dryer, 2025). Briefly, a confluent layer of MPC‐5 precursor cells was plated onto the inserts of Corning 12 mm transwell plates (0.4 μM pore size for the transwell inserts, catalog number 3460) and differentiated into a podocyte phenotype. At the end of various treatments described below, albumin–fluorescein isothiocyanate (FITC‐albumin) (#A9771) obtained from Millipore Sigma (St. Louis, MO, USA) was dissolved in normal culture media at a concentration of 1 mg/mL and was introduced onto the cis side of the transwell chamber for 3 h at 37°. We have previously observed that this duration allowed for reproducible albumin permeation measurements (Kim & Dryer, 2025). Media on the trans side of the chamber was collected, and FITC‐albumin was quantified on a fluorescence plate reader (excitation at 490 nm and emission at 530 nm).

### 2.3 Immunoblot, siRNA, ROS, and ELISA analyses

Podocyte lysates were separated on 10% SDS–PAGE gels and transferred to nitrocellulose membranes (Invitrogen, catalog number IB33001X3). Membranes were incubated with primary antibodies overnight at 4°C. Secondary antibodies used to probe blots were HRP‐linked anti‐rabbit IgG Antibody (catalog number 7074) and HRP‐linked anti‐mouse IgG (catalog number 7076), both from Cell Signaling Technology Inc. and both used at a dilution of 1:5000. The following primary antibodies were used (all at 1:1000 dilution unless otherwise noted): phospho NFκB p65 (3033S), NF κB p65 (4764S), phospho p44/42 MAPK (Erk1/2) (9101S), p44/42 MAPK (Erk1/2) (9102S), cleaved caspase 3 (9661S), and MyD88 (4283S) (Cell Signaling Technology, Danvers, MA, USA). Antibodies against synaptopodin (sc 515842), nephrin (sc 376522), WT‐1 (sc 192), and podocin (sc 518088) were obtained from Santa Cruz Biotechnology (Santa Cruz, CA, USA). An antibody against β actin (ZMS1004; MilliporeSigma) was used at 1:5000 dilution. Protein bands were visualized using a SuperSignal™ West Pico PLUS Chemiluminescent Substrate (Thermo Fisher Inc., catalog number 34580), and signal intensities of the bands at the appropriate molecular weight were quantified using ImageJ software (Bethesda, MD, USA). For transient knockdown experiments, podocytes were transfected with siRNA targeting MyD88 (sc 35,978) or non‐targeting control siRNA (sc 37007) (Santa Cruz Biotechnology) using Lipofectamine™ 3000 (Thermo Fisher Scientific, Waltham, MA, USA). Cytosolic reactive oxygen species (ROS) levels were measured using a fluorometric assay based on the cell permeable probe 2′,7′ dichlorodihydrofluorescein diacetate (Cell Biolabs Inc., San Diego, CA, USA). Annexin V levels were measured by ELISA using the Annexin V FITC Apoptosis Detection Kit I (556,547; BD Biosciences, Franklin Lakes, NJ, USA). Culture supernatants from podocytes grown in standard dishes were collected, centrifuged to remove debris, and analyzed for nephrin shedding using the Mouse Nephrin ELISA Kit (NBP2 76750; Novus Biologicals, Centennial, CO, USA) as described previously (Kim & Dryer, 2025).

### 2.4 Drugs

CRX527 (tlrl crx527) was purchased from InvivoGen (San Diego, CA, USA). TAK 242 (614316), TEMPOL (581500) were obtained from MilliporeSigma (Burlington, MA, USA). JSH 23 (S7351) and PD98059 (S1177) were purchased from Selleckchem (Houston, TX, USA). Cycloheximide (2112) was obtained from Cell Signaling Technology (Danvers, MA, USA), and Batimastat (HY 13564) was purchased from MedChemExpress (Monmouth Junction, NJ, USA). The TRPC6 inhibitor (4‐[[(1R,2R)‐2‐[(3R)‐3‐amino‐1‐piperidinyl]‐2,3‐dihydro‐1H‐inden‐1‐yl]oxy]‐3‐chlorobenzonitrile dihydrochloride) (SAR7334) was obtained from Cayman Chemical (Ann Arbor, MI, USA, catalog number 28292).

### 2.5 Statistical analyses

In experiments in which multiple comparisons were made between groups, data were analyzed by one‐way or two‐way ANOVA and Dunnett's post hoc test (α = 0.05). The homogeneity of variances in all of these tests was confirmed using the Brown‐Forsythe test. In experiments in which a single experimental group was compared against a control group, data were analyzed by Student's unpaired *t*‐test, with *p* < 0.05 regarded as significant. Statistical analyzes were carried out using Graphpad Prism v10™ software. Scatter plots in figures show results of independent measurements as points along with mean and SD.

## 3 RESULTS

### 3.1 Effects of TLR4 activation on the albumin permeability of confluent monolayers of immortalized mouse podocytes

In initial experiments, confluent layers of podocytes were cultured on the *cis* side of a transwell chamber in the presence and absence of the selective TLR4 agonist CRX527 for various periods of time. CRX527, a synthetic aminoalkyl glucosaminide 4‐phosphate compound, is a structural analog of lipid A, the toxic component of LPS (Legat et al., 2010). Control cells were exposed to media and vehicle. Following agonist exposure, treated cells and control cells were returned to normal media and FITC‐albumin was introduced into the *cis* side of the chamber allowed to diffuse across the transwell for 3 h, and then quantified in media collected from the *trans* side of the chamber. We observed that application of CRX527 (0.7 μM) for 1 or 24 h evoked a significant increase in FITC‐albumin movement across the podocyte layer compared to controls, hereafter referred to as an increase in *P*_Alb_ (Figure 1a). Previous studies carried out in monocyte‐derived macrophages have shown that this concentration of CRX527 is in the middle range of the concentration‐response curve (Stöver et al., 2004), which also appears to be the case in podocytes (see Figure S1). The effects of both the 1 or 24‐h exposures to CRX527 on *P*_Alb_ were completely blocked by concurrent exposure to TAK242 (1 μM), a selective antagonist of TLR4 that does not bind to other members of the Toll‐like receptor family (Takashima et al., 2009). TAK242 by itself for 1 or 24 h had no effect on *P*_Alb_ (Figure 1b). Notably, the increase in *P*_Alb_ could no longer be detected if a 72‐h recovery period in normal media was allowed prior to measurement of albumin flux (Figure 1c), indicating that the effect of a 24‐h exposure to CRX527 is reversible.

**FIGURE 1:**
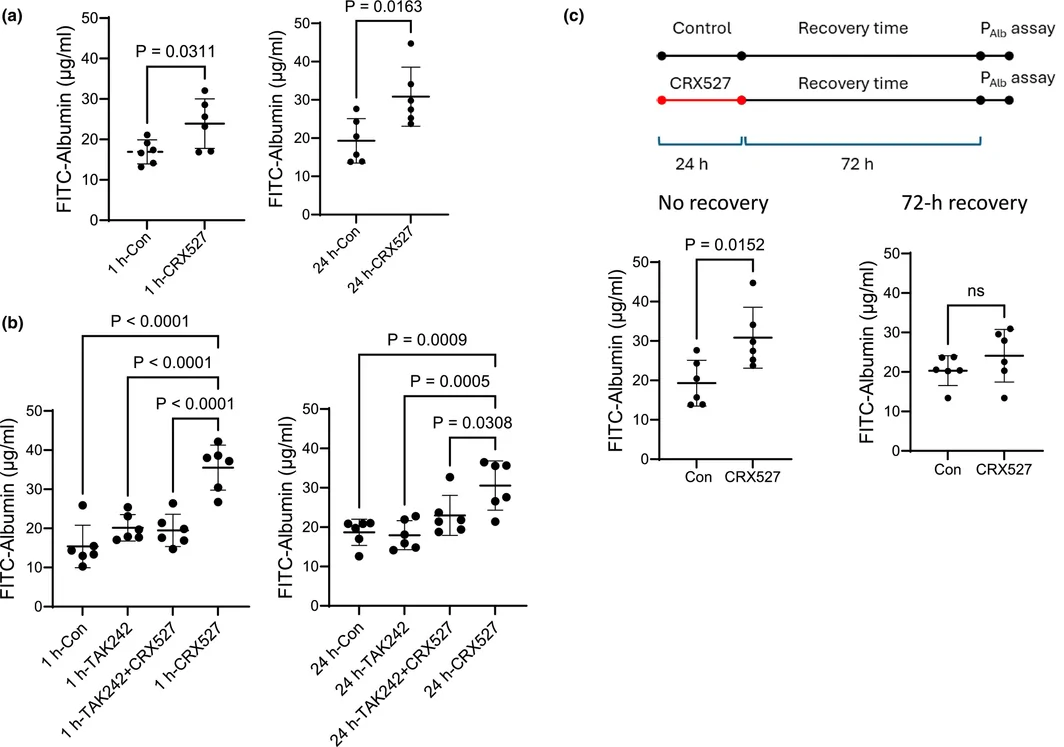
TLR4 activation increases *P*_Alb_ across a confluent podocyte monolayer in transwell assays. Cells were treated with the TLR4 agonist CRX527 (0.7 μM) for 1 h or 24 h, prior to estimating *P*_Alb_ by quantifying cumulative FITC‐albumin movement across the monolayer during a 3 h period commencing immediately after the termination of agonist treatment. (a) CRX527 treatments of 1 h (left) or 24 h (right) evoked an increase in *P*_Alb_. *p* values indicate significant difference as assessed by unpaired *t*‐test. (b) The effects of CRX527 treatment for 1 h or 24 h were blocked by the TLR4 antagonist TAK242 (1 μM). TAK242 by itself had no effect on *P*_Alb_. Data in these experiments were analyzed by one‐way ANOVA followed by Dunnett's post hoc test. *p* values for selected post hoc comparisons are indicated in the graphs. (c) The effects of a 24‐h exposure to CRX527 were readily detectable when *P*_Alb_ was measured immediately after the end of agonist exposure (left). The increase in *P*_Alb_ could not be detected if cells were returned to control media for 72 h prior to measurement of *P*_Alb_ (right). In these graphs, points indicate individual measurements, horizontal bars indicate the mean of the group, and error bars indicate SD.

### 3.2 TLR4 activation for 24 h induces shedding of nephrin ectodomains

Under normal conditions, slit diaphragms connect highly specialized domains of adjacent foot processes through adhesive interactions mediated in part by nephrin (Ruotsalainen et al., 1999). In glomerular diseases, nephrin appears to be decreased in regions where foot process effacement is present but is maintained in areas where foot process ultrastructure is preserved (Huh et al., 2002). Using a commercial ELISA assay, we observed that a 24‐h treatment with CRX527 caused a marked increase in soluble nephrin measured in the surrounding culture medium (Figure 2a). This was not due to cells or cell fragments that have detached from the substrate, as any of those were pelleted by centrifugation prior to assay of the supernatant. Previous studies have shown that nephrin ectodomains can be shed from podocytes (Collino et al., 2008; Doublier et al., 2001; Kim & Dryer, 2025) through actions of various matrix metalloproteinases (MMPs) (Tan et al., 2019). Soluble nephrin release was not detected in cells treated with CRX527 for 1 h (Figure 2b) even though this was sufficient to increase *P*_Alb_ (Figure 1a). Soluble nephrin release evoked by a 24‐h exposure to CRX527 was inhibited by TAK242, and also by concurrent exposure to Batimistat (BB‐94) (Figure 2c), a broad‐spectrum inhibitor of MMPs and sheddase enzymes in the A Disintegrin And Metalloprotease (ADAM) family. This observation suggests that nephrin ectodomains were shed into the media due to proteolytic cleavage. Nephrin ectodomain shedding was also blocked by concurrent exposure to CRX527 and cycloheximide (CHM), an inhibitor of protein synthesis (Figure 2d). Immunoblot analysis showed that the total abundance of podocin, synaptopodin, and nephrin was not reduced in cells treated with CRX527 for 24 h, which would not be expected if any significant amount of nephrin in the media was a consequence of cell detachment (Figure 3a–c). By contrast, Wilm's tumor‐1 (WT‐1), a transcription factor that promotes a mature podocyte phenotype, was decreased following a 24‐h treatment with CRX527 (Figure 3d).

**FIGURE 2:**
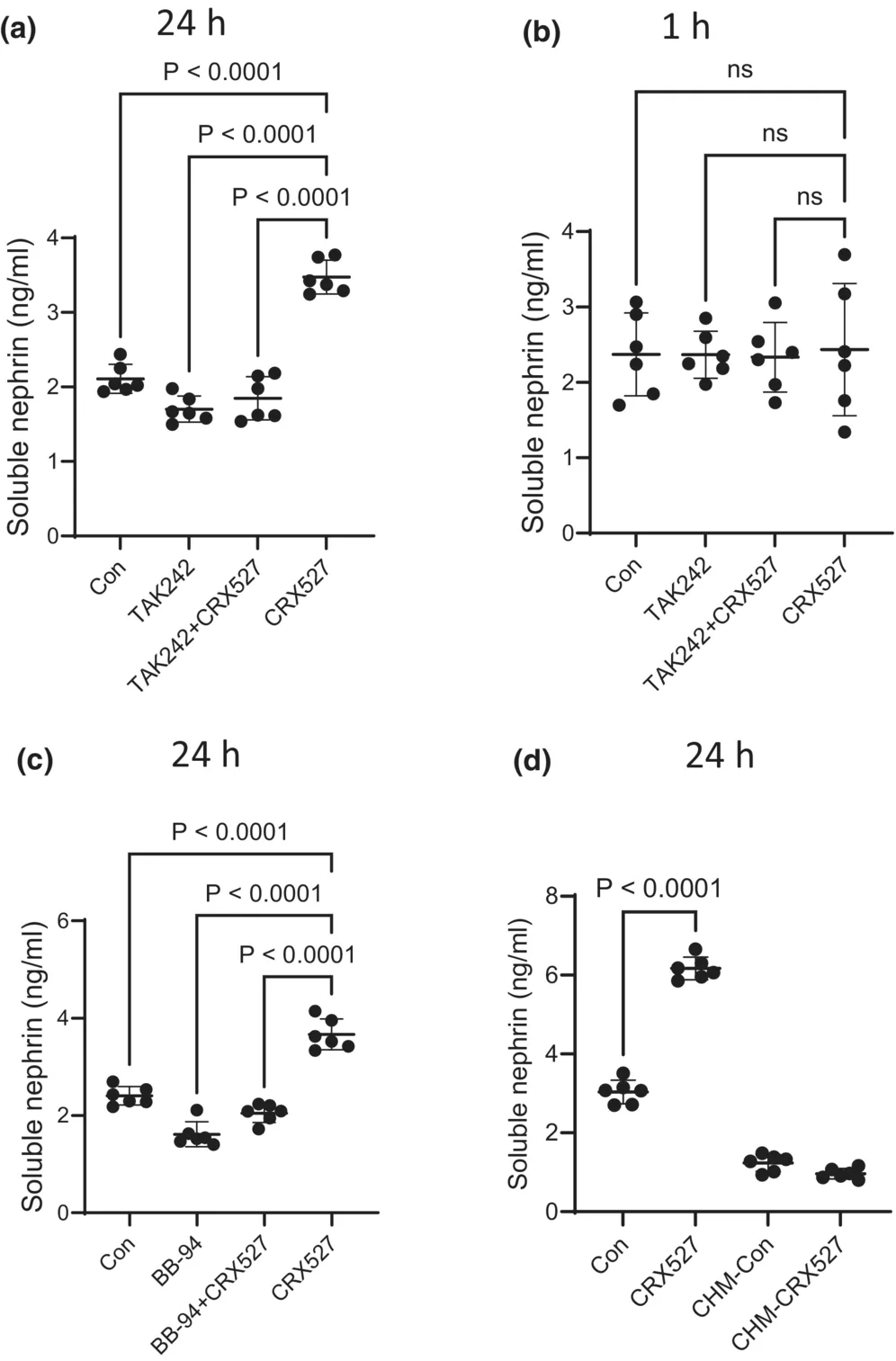
TLR4 activation increases shedding nephrin ectodomains from cultured podocytes. Cells were treated with CRX527 (0.7 μM) for various lengths of time in the presence or absence of various inhibitors, as indicated. (a) CRX527 exposure for 24 h increased soluble nephrin detected in the culture media, and this effect was inhibited by TAK242. (b) CRX527 treatment for 1 h was not sufficient to induce nephrin shedding. (c) The effects of a 24 h exposure to CRX527 were blocked by BB‐94, a broad‐spectrum inhibitor of matrix metalloproteinases. (d) The effects of CRX527 were also blocked by concurrent exposure to cycloheximide (CHM). An inhibitor of protein synthesis. In these graphs, points indicate individual measurements, horizontal bars indicate the mean of the group, and error bars indicate SD.

**FIGURE 3:**
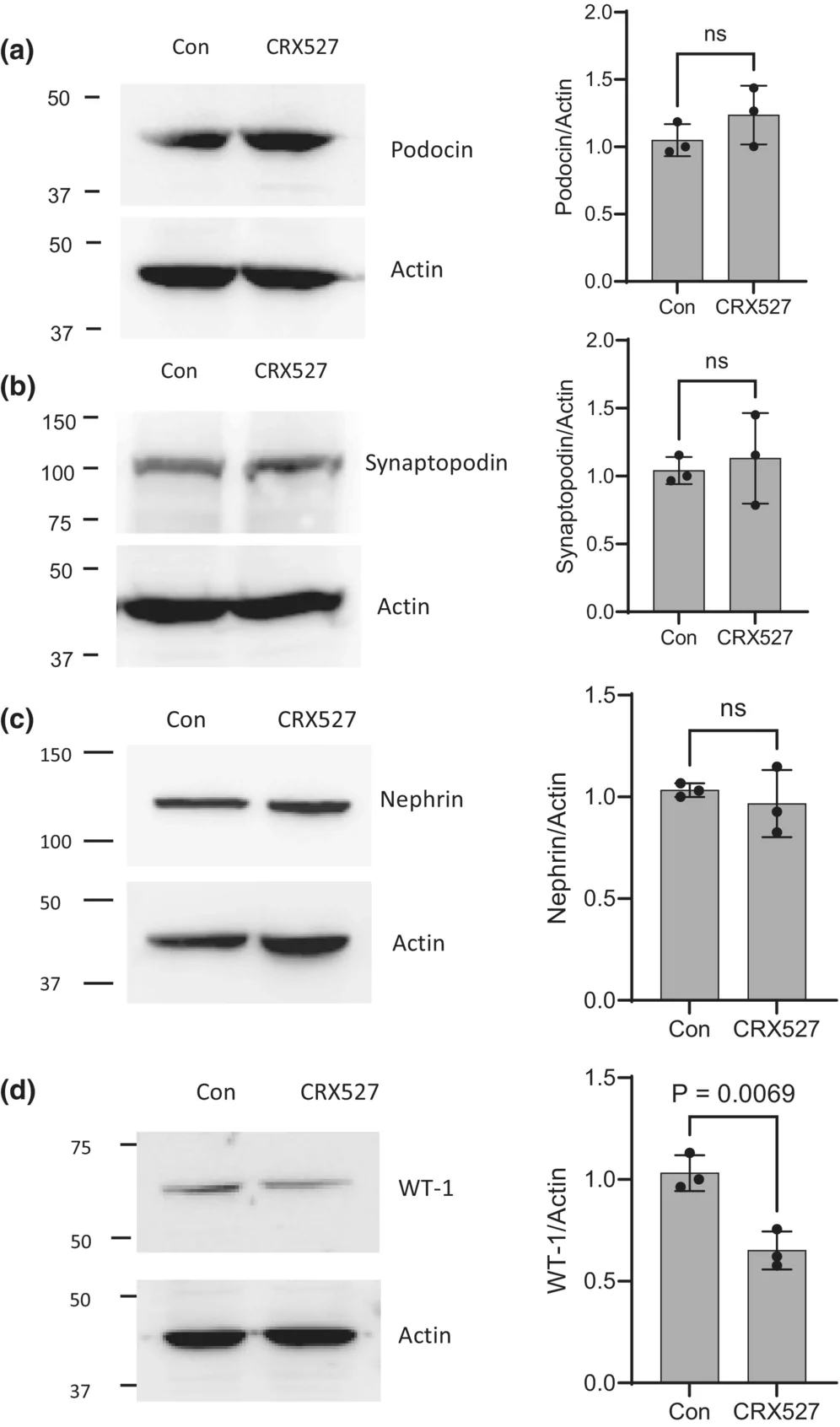
Exposure to CRX527 for 24 h does not affect the total amounts of podocin (a), synaptopodin (b), or nephrin (c) as analyzed by immunoblot in cultured podocytes. However, CRX527 induced a significant decrease in abundance of the podocyte transcription factor WT‐1 (d). Points on the bar graphs indicate a replicate of the experiment.

### 3.3 More sustained TLR4 activation induces podocyte apoptosis

A 72‐h continuous exposure to CRX527 caused marked increases in cleaved caspase‐3 (CC3) as measured by immunoblot (Figure 4a) and increases in annexin V measured by ELISA (Figure 4b), suggesting that more sustained continuous TLR4 activation triggers processes that eventually lead to apoptotic cell death. The increase in CC3 was blocked by concurrent exposure to TAK242 (Figure 4a). CRX527 treatment for 24 h was not sufficient to cause detectable changes in CC3 abundance (Figure 4c).

**FIGURE 4:**
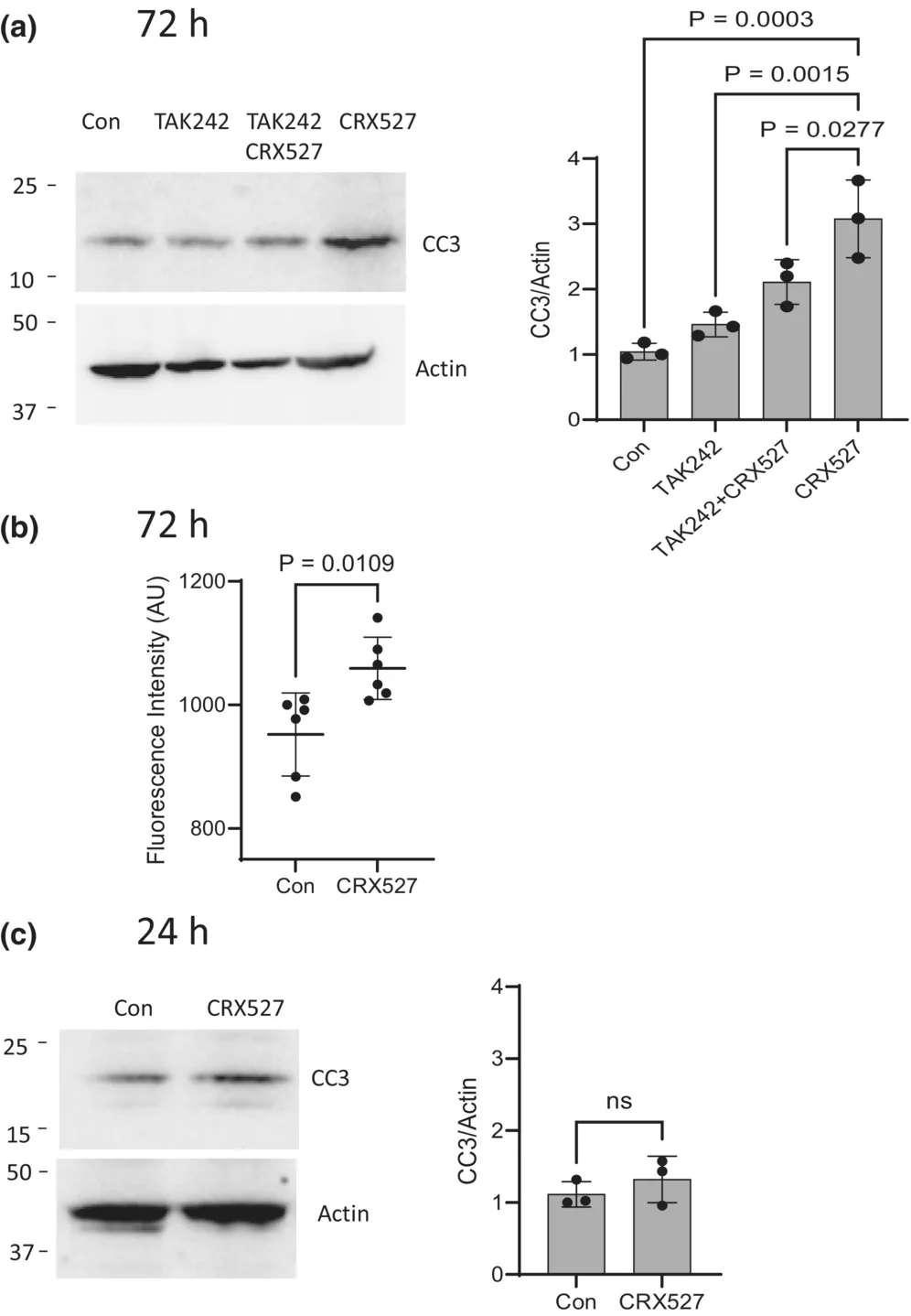
Continuous TLR4 activation for 72 h induces apoptosis in podocytes. Apoptosis was assayed by immunoblot analysis of cleaved caspase‐3. (a) Immunoblot analysis of cleaved caspase‐3 (CC3) indicating that CRX527 exposure for 72 h induced apoptosis and this effect was inhibited by TAK242. (b) CRX527‐evoked increase in apoptosis was also seen by using ELISA measurement of Annexin V. In these graphs, points indicate individual measurements, horizontal bars indicate the mean of the group, and error bars indicate SD. (c) CRX527 treatment for 24 h was not sufficient to cause cleavage of caspase‐3. Immunoblots in A and Cshow representative assays, and densitometric quantification is shown to the right, with each point representing a replicate of the experiment.

### 3.4 Intracellular signal cascades activated by TLR4 in podocytes

Application of TLR4 causes activation of multiple intracellular pathways, depending on the duration of CRX527 exposure. In this study we have focused on cytosolic ROS generation, activation of Erk MAP kinase, and activation of NFκB. Each of these intracellular messages increased following TLR4 activation in immune cells, albeit with different time courses. Application of CRX527 evoked a rapid burst of Erk activation as assessed by immunoblot analyses using antibodies that detect phosphorylated and total forms of Erk1/2. Figure 5a shows Erk activation evoked by a 30‐m exposure to CRX527. This effect was blocked by TAK242. Application of CRX527 for either 1 or 24 h also evoked an increase in cytosolic ROS measured using a fluorometric assay, and this effect was blocked by TAK242 (Figure 5b,c). Finally, a 24‐h exposure to CRX527 also resulted in activation of NFκB, seen as an increase in phospho‐p65 as measured by immunoblot analysis that was blocked by TAK242 (Figure 5d). In contrast to ROS or Erk, a 1‐h exposure to CRX527 was not sufficient to cause activation of NFκB (Figure S2). In addition, activation of NFκB was inhibited by siRNA knockdown of MyD88 (Figure S2), an adapter protein that connects TLR4 receptors to various downstream transducing enzymes (Keogh & Parker, 2011).

**FIGURE 5:**
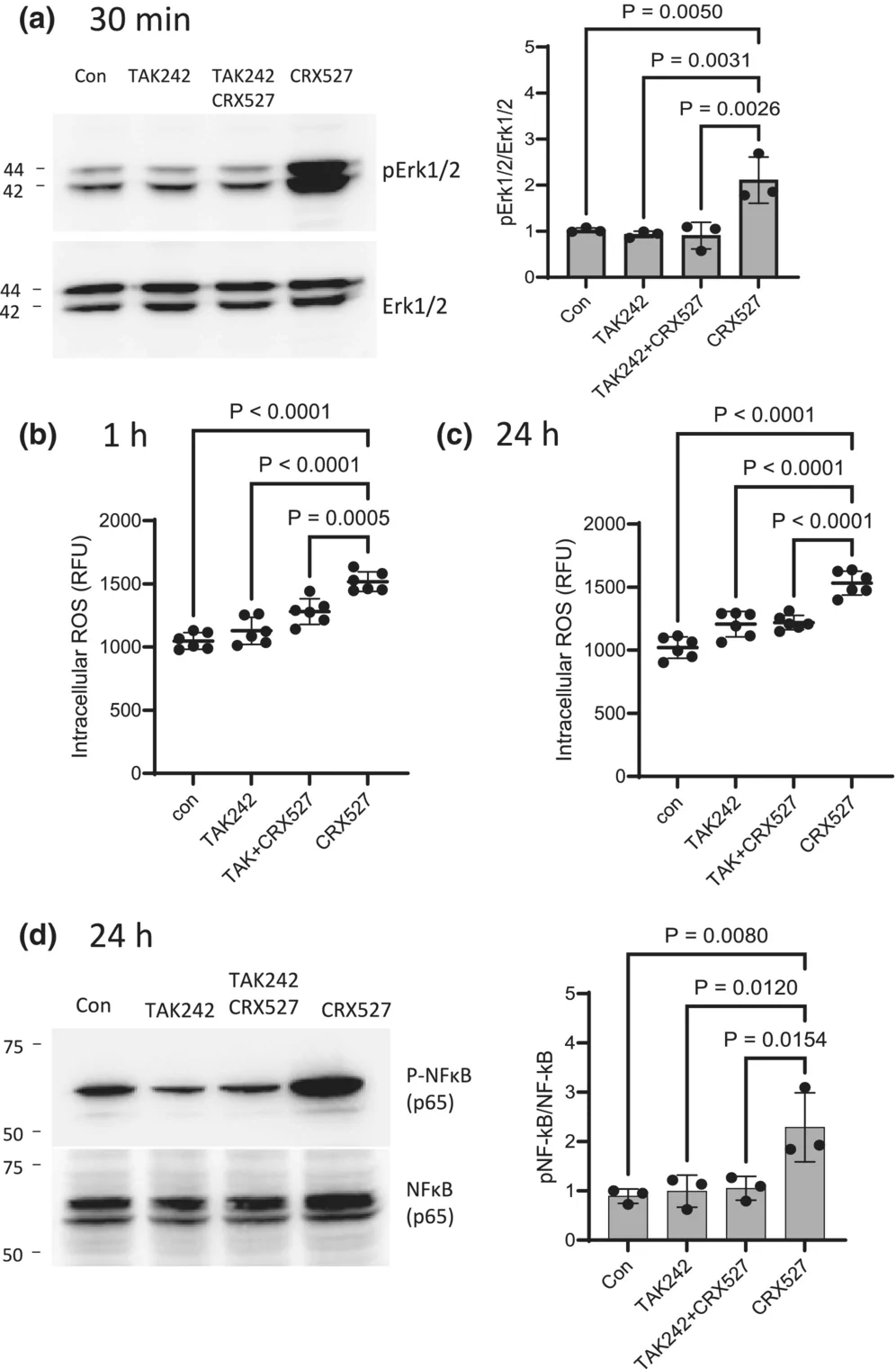
Intracellular signals engaged following TLR4 activation for various periods of time in podocytes. (a) CRX527 exposure for 30 m causes activation of Erk1/2, assayed by immunoblot analysis of phosphorylated (active) and total amounts detected in podocyte lysates. This effect was blocked by TAK242. Representative blots are shown to the left, and densitometric analyses are to the right. (b) CRX527 treatment for 1 h was sufficient to induce a detectable increase in ROS measured by a fluorometric assay, and this effect was blocked by TAK242. (c) Increased ROS production was sustained during a 24‐h exposure to CRX527. (d)CRX527 exposure for 24 h caused activation of NFκB, seen in immunoblot analyses as an increase in phosphorylated p65. This effect was blocked by TAK242. In these graphs, points indicate individual measurements, horizontal bars indicate the mean of the group, and error bars indicate SD.

### 3.5 Role of intracellular signal cascades in driving functional responses to TLR4 activation of various durations in podocytes

The previous experiments identified three distinct functional responses to TLR4 activation in podocytes that vary with the duration of agonist exposure: (1) Rapid increases in *P*_Alb_; (2) nephrin ectodomain shedding; and (3) apoptosis. They also demonstrated very rapid activation of Erk1/2, a relatively rapid increase in cytosolic ROS, and activation of NFκB that occurs after a longer delay. Therefore, we conducted experiments to determine what, if any, role these intracellular signals play in the three functional outputs. The increase in *P*_Alb_ observed at 1 h was at least partially reduced in the presence of either TEMPOL, a membrane‐permeable small molecule that quenches cytosolic ROS regardless of the source (Figure 6a), or by PD98059, a MEK1 inhibitor that blocks activation of Erk1/2 signaling (Figure 6b). NFκB activation was not detected at the 1‐h time point in podocytes, and the increase in *P*_Alb_ persisted in cells concurrently treated for 24 h with CRX527 and JSH‐23, an agent that inhibits NFκB signaling by blocking its translocation to nuclei (Shin et al., 2004) (Figure 6c).

**FIGURE 6:**
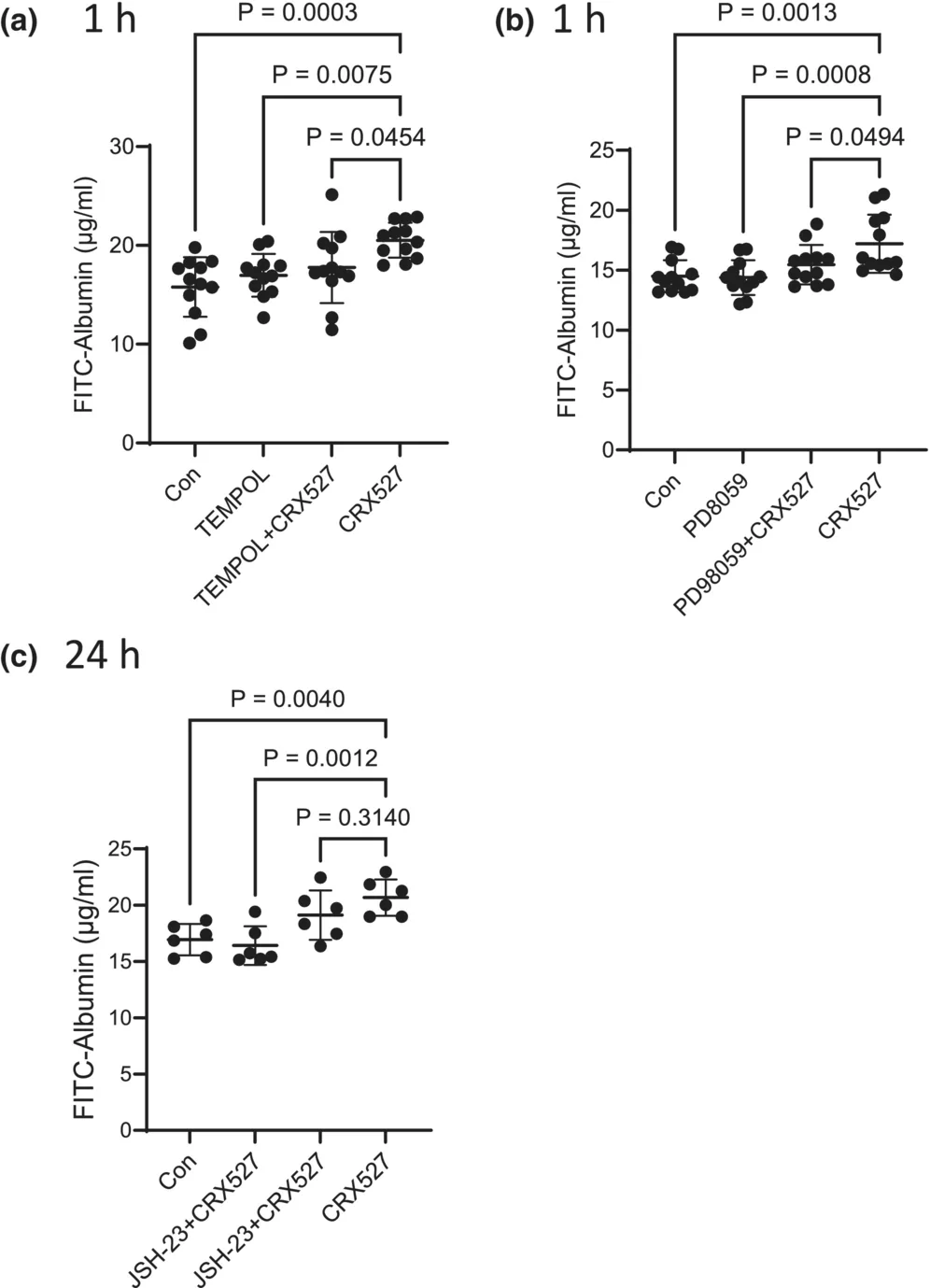
Role of intracellular signaling cascades in TLR4‐mediated increase in *P*_Alb_. CRX527 exposure for 1 h causes increase in *P*_Alb_ that was reduced by concurrent exposure to the ROS quencher TEMPOL (a) or by the MEK1 inhibitor PD98059 (b), which blocks activation of Erk1/2. (c) CRX527 exposure for 24 h caused an increase in *P*_Alb_ that persisted in the presence of JSH‐23, an agent that blocks activation of NFκB signaling. In these graphs, points indicate individual measurements, horizontal bars indicate the mean of the group, and error bars indicate SD.

In a similar set of experiments, we examined TLR4‐mediated nephrin ectodomain shedding measured after a 24‐h CRX527 treatment (since it does not occur following a 1‐h exposure). The increase in nephrin shedding was also blocked by TEMPOL (Figure 7a), but it persisted in the presence of either the ROS quencher TEMPOL (Figure 7b) or the NFκB inhibitor JSH‐23 (Figure 7c).

**FIGURE 7:**
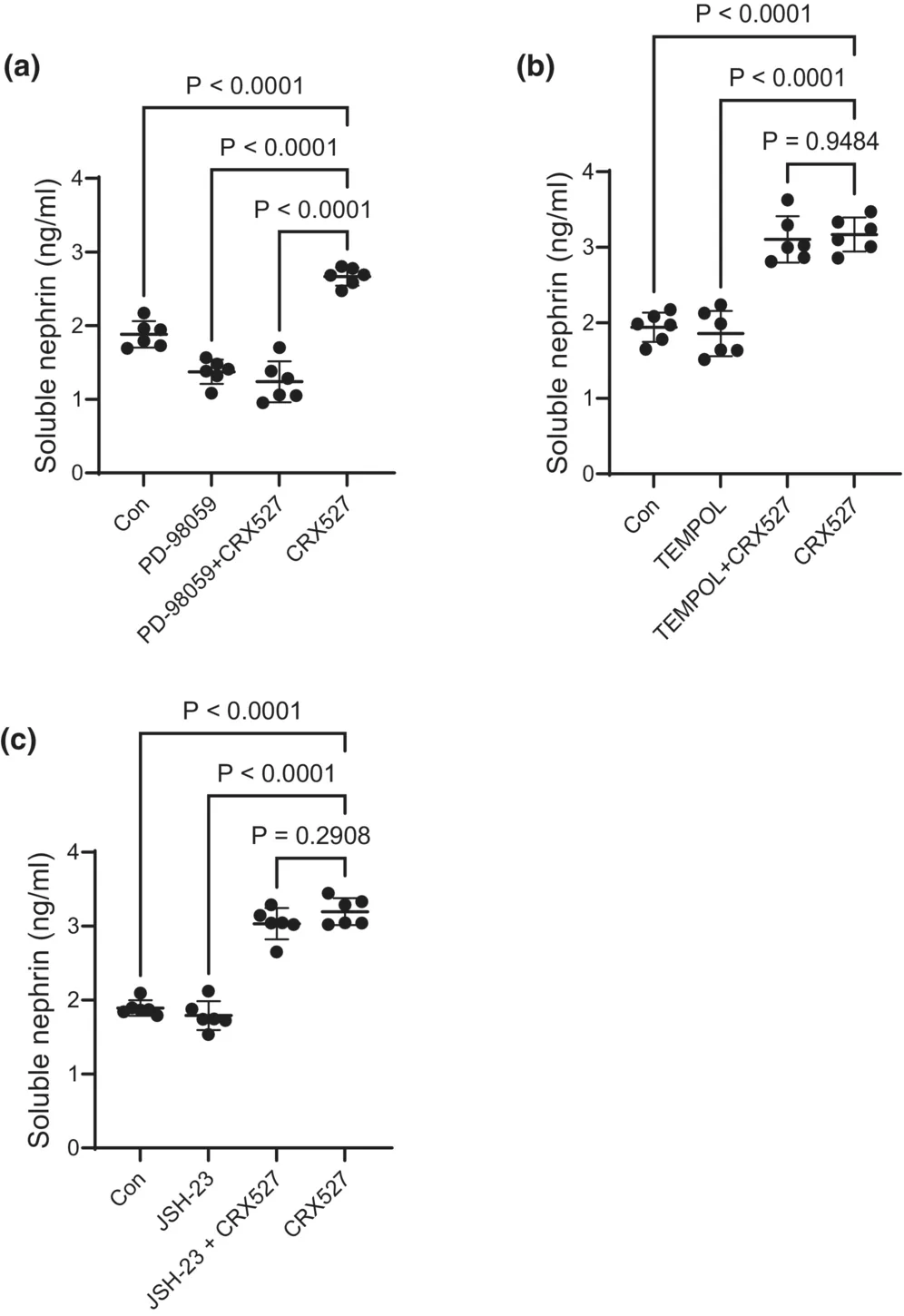
Role of intracellular signaling cascades in TLR4‐mediated nephrin ectodomain shedding. CRX527 exposure for 24 h caused an increase in nephrin shedding that was blocked by the Erk pathway inhibitor PD98059 (a). By contrast, this response persisted in the presence of the ROS quencher TEMPOL (b), and in the presence of JSH‐23, an agent that blocks activation of NFκB signaling (c). In these graphs, points indicate individual measurements, horizontal bars indicate the mean of the group, and error bars indicate SD. Data were analyzed using one‐way ANOVA followed by Dunnett's post hoc test.

Finally, we observed that apoptosis, seen as an increase in CC3 following a 72‐h exposure to CRX527 that persisted in the presence of PD98059 (Figure 8a), was completely blocked by concurrent quenching of ROS using TEMPOL (Figure 8b), and also by inhibition of NFκB signaling using JSH‐23 (Figure 8c). In other words, each of the three functional responses has a different time course, and a distinct dependence on the three intracellular signaling pathways examined here.

**FIGURE 8:**
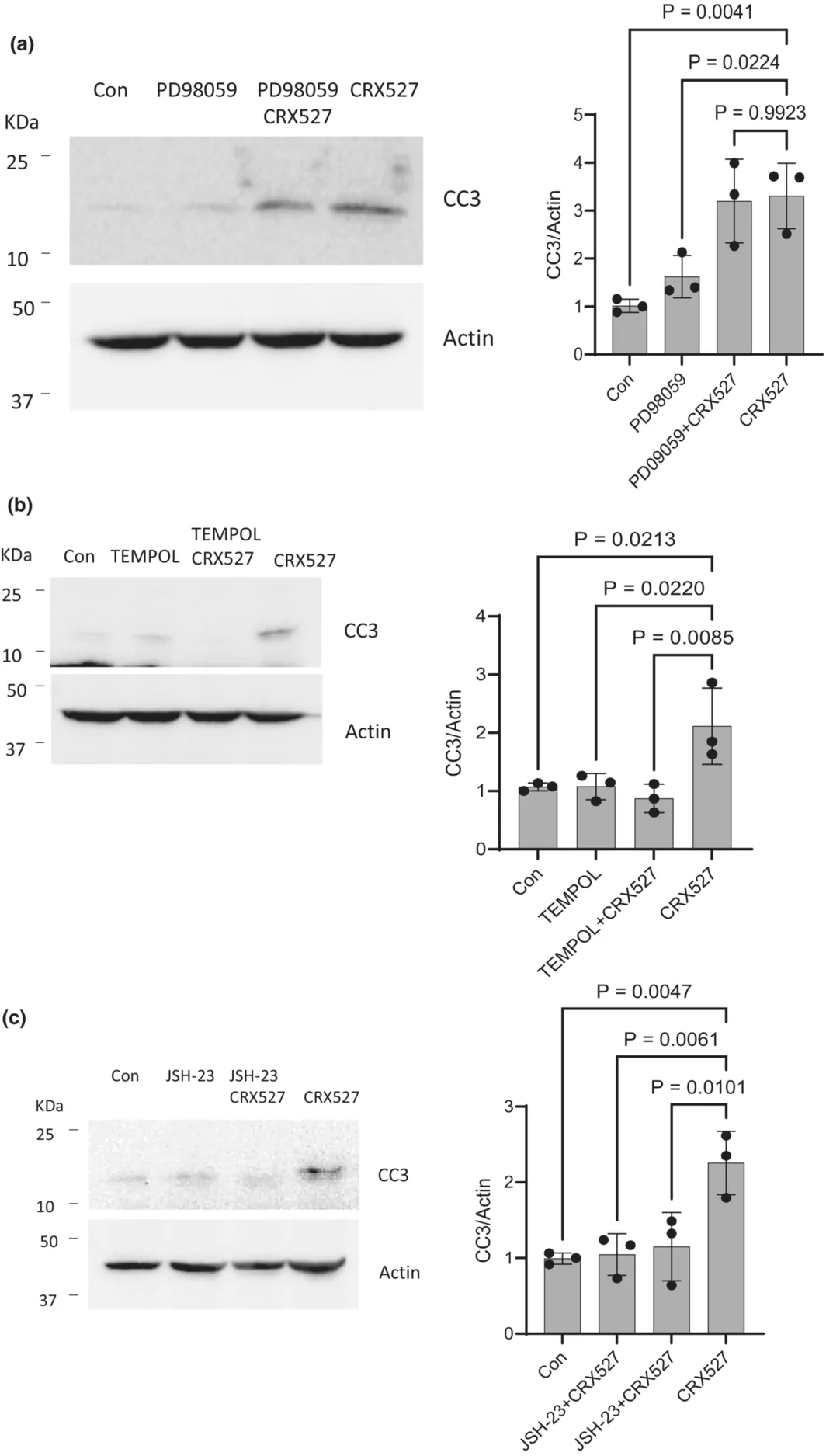
Role of intracellular signaling cascades in TLR4‐mediated apoptosis in podocytes. CRX527 exposure for 72 h causes an increase in apoptosis as indicated by an increase in cleaved caspase‐3. This effect persisted in the presence of the Erk pathway inhibitor PD98059 (a), but apoptosis was reduced or blocked in the presence of the ROS quencher TEMPOL (b), and in the presence of the NFκB pathway inhibitor JSH‐23 (c). In these graphs, points on the bar graphs indicate measurements from different immunoblots, horizontal bars indicate the mean of the group, and error bars indicate SD.

## 4 DISCUSSION

TLR4 receptors are expressed in podocytes and appear to contribute to glomerular injury in in vivo disease models (Banas et al., 2008; Li et al., 2025; Ma et al., 2014) but TLR4 signaling mechanisms and their functional effects in podocytes have not been extensively characterized. The present study has shown that TLR4 produces multiple effects in cultured podocytes that depend on the duration of agonist exposure. Thus, TLR4 agonists induce a very rapid but transient activation of Erk MAP kinase, along with a subsequent increase in the generation of ROS. More sustained TLR4 activation results in production of active NFκB. TLR4 agonists can also induce a fully reversible increase in P_Alb_ across a podocyte monolayer, and this can be detected in as little as 1 h. ROS and Erk activation are necessary for this response. Continuous activation of TLR4 for 24 h induces shedding of nephrin ectodomains mediated by metalloproteases. This response also requires Erk activation, but in contrast to the increase in P_Alb_, does not require increases in ROS. It also persists when NFκB activation is blocked. In addition, nephrin ectodomain shedding is not accompanied by a change in the total abundance of either podocin or synaptopodin, key proteins of podocyte foot processes, which along with the methods used in these assays, argues against cell detachment as an explanation for the observed responses. Somewhat surprisingly, total nephrin did not decrease either, but there was a decrease in WT‐1, a transcription factor that contributes to the fully mature podocyte phenotype. Finally, continuous TLR4 activation for 72 h induces apoptosis, and ROS and NFκB activation are both necessary for this response. These results are summarized schematically in Figure 9.

**FIGURE 9:**
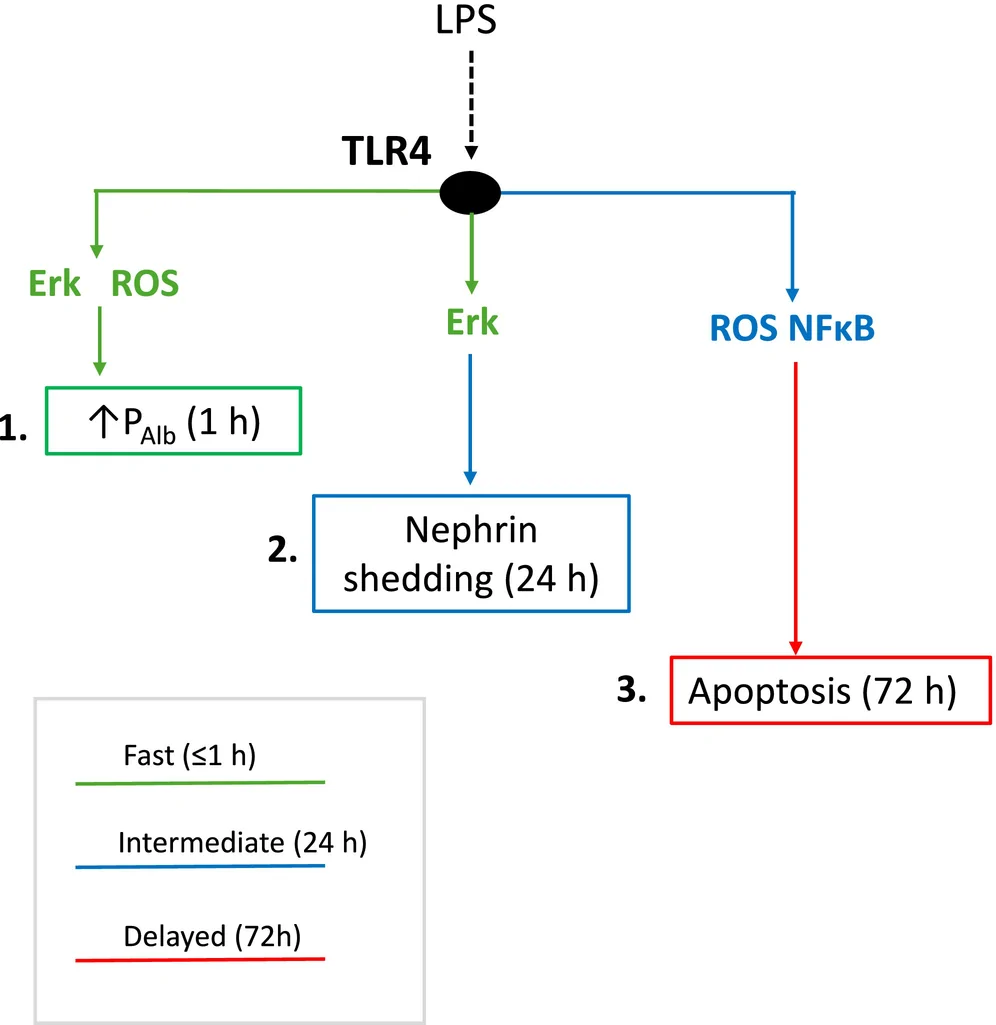
Schematic diagram summarizing three different functional consequences of TLR4 signaling in podocytes, their relative time course as estimated by the assays used in this study, and the signaling pathways required to observe each functional response, based on pharmacological analyses.

Throughout this study we utilized a synthetic agonist CRX527 to induce specific activation of TLR4 and have demonstrated that its effects are blocked by the selective and structurally dissimilar TLR4 antagonist TAK242. The concentration used here is close to the EC50 reported previously, and we have carried out experiments that show that 0.7 μM does not produce maximum effects in podocytes (Figure S1). The use of in vitro experimental designs with only a single cell type ensured that the effects were a consequence of TLR4 receptors on podocytes themselves. While it is certainly possible that some of the responses here reflect paracrine signaling between neighboring podocytes (Banas et al., 2008), the cascades observed here are initiated by TLR4 receptors on podocytes (since no other cell type is present). Previous studies have shown that injection of the TLR4 agonist LPS can induce a reversible proteinuria in mice (Lorenzen et al., 2008; Reiser et al., 2004) but those responses could have been secondary to its well‐known effects on other cell types, for example by stimulating TNFα or IL‐6 secretion from myeloid cells (Rossol et al., 2011).

The canonical signaling pathway from TLR4 to NFκB has been observed previously in podocytes (Hu et al., 2022). In the present study we have expanded on those observations by showing that TLR4 signaling is pleiotropic in podocytes and acts over a range of time courses, and we have shown that these intracellular signals are differentially coupled to functional output effects in podocytes. TLR4 signaling in immune cells has been studied far more extensively, and its pleiotropic nature is apparent at the earliest stages (Luo et al., 2025). In those cells, TLR4 in the plasma membrane causes activation of the TRAP‐MyD88 pathway, which subsequently drives activation of NFκB. Following internalization, TLR4 in endosomes initiates the TRAM‐TRIF pathway, leading to the activation of IRF3, the production of type I IFNs, and a late wave of NFκB activation. In immune cells, TLR4 can drive increases in cytosolic ROS and activation of the metalloproteinase ADAM17 through a TRIF‐pathway independent of MyoD (Thorp et al., 2011), and this in turn can lead to shedding of a variety of membrane protein ectodomains. It seems likely that similar processes are occurring in podocytes, at least following 24 h of TLR4 activation (see Figure S2).

LPS is not the only type of molecule that can induce a relatively rapid and reversible increase in glomerular permeability. Notably, infusion of angiotensin II (Ang II) and atrial natriuretic peptide can induce an increase in glomerular permeability to macromolecules such as Ficoll (Axelsson et al., 2012) or albumin (Königshausen et al., 2016) that could be observed in 15–60 min, even in the absence of increases in blood pressure. The effect of Ang II is mediated by AT1 receptors and is associated with an increase in nephrin endocytosis mediated by interactions between nephrin and β‐arrestin‐2 (Königshausen et al., 2016, 2024). This effect also requires activation of Erk/1/2, similar to the increase in ectodomain shedding observed here. An interesting aspect of Erk1/2 activation by Ang II is that it occurs through complex pathways that include *trans*‐activation of epidermal growth factor receptors (EGFRs) (Forrester et al., 2018). TLR4 agonists can also induce trans‐activation of EGFRs as a consequence of increased ROS generation (Cho et al., 2016). It bears noting that the processes of endocytosis and ectodomain shedding of single‐pass membrane proteins are often linked, albeit in complex ways. Thus, shedding of ectodomains by metalloproteinases can facilitate endocytosis of the remaining trans‐membrane domains and cytoplasmic tails (Hayashida et al., 2010), and endocytosis can lead to recycling of surface proteins to membrane zones where shedding is more likely to occur (Tosetti et al., 2021). It is certainly possible that nephrin undergoes similar dynamics, as both processes would lead to an increase in the number of “large pores” at slit diaphragms that would have an elevated permeability to macromolecules such as albumin (Axelsson et al., 2012).

One feature of Ang II signaling in podocytes is the activation of cell surface Ca^2+^‐permeable TRPC6 channels (Anderson et al., 2014). We have recently reported that sustained (24‐h) activation of TRPC6 channels leads to shedding of nephrin ectodomains from cultured podocytes that is dependent on metalloproteinases, similar to the responses observed here in response to TLR4 activation (Kim & Dryer, 2025). However, direct TRPC6 activation did not cause increases in P_Alb_ at either 1 or 24 h. Here we observed that the TLR4 agonist CRX527 continued to cause significant nephrin shedding in the presence of SAR‐7334 (Figure S3), a drug that causes complete and relatively selective inhibition of TRPC6 channels in podocytes (Dryer et al., 2019). Consequently, TRPC6 does not appear to be an essential component of TLR4‐induced nephrin shedding in podocytes.

The most important limitation of this study is that the experiments were carried out entirely in vitro, and the increases in *P*_Alb_ may not entirely recapitulate acute changes in glomerular filter function that occur in vivo, although it should be emphasized that these cells form junctions in vitro with ultrastructural features similar to slit diaphragms (Hunt et al., 2005; Reiser et al., 2000; Shankland et al., 2007), and administration of LPS certainly induces proteinuria (Lorenzen et al., 2008; Reiser et al., 2004). An important advantage of the approach used here is that it avoided secondary effects caused by actions of TLR4 agonists on other cell types and allowed for a more precise analysis of TLR4 signal transduction. It is possible that the concentrations of CRX527 used in these experiments may be producing a greater effect than would be evoked in vivo by DAMP and PAMP agonists of TLR4, which could enhance or accelerate processes such as apoptotic cell death observed here. Finally, the processes and pathways examined here were not intended to be exhaustive, and it is quite likely TLR4 activation produces other effects not observed here. Nevertheless, these observations support the idea that at least a portion of the LPS‐induced proteinuria reported previously was due to direct effects on podocytes, and they provide a plausible mechanistic hypothesis to explain common and benign transient proteinurias.

## AUTHOR CONTRIBUTIONS

**Eun Young Kim:** Data curation; formal analysis; investigation. **Stuart E. Dryer:** Conceptualization; funding acquisition; project administration; supervision.

## FUNDING INFORMATION

Supported by NIH Grant R01‐DK104708 (to SED).

## CONFLICT OF INTEREST STATEMENT

The authors declare no conflicts of interest.

## ETHICS STATEMENT

This study does not report experiments carried out on animals, human subjects, or human fetal tissue.

## Supporting information


**Figure S1.** Effects of CRX527 on ROS generation after 24 h. The experiments used in the body of the MS utilized 0.7 μM.
**Figure S2.** MyD88 and NfKB activation at different time points (A, B) and at 24 h and after siRNA knockdown of MyD88 (C).
**Figure S3.** Significant nephrin ectodomain shedding persists following blockade of TRPC6 channels.

## Data Availability

Data are available upon request.

## References

[phy271147-bib-0001] Anderson, M. , Roshanravan, H. , Khine, J. , & Dryer, S. E. (2014). Angiotensin II activation of TRPC6 channels in rat podocytes requires generation of reactive oxygen species. Journal of Cellular Physiology, 229(4), 434–442. 10.1002/jcp.24461 24037962

[phy271147-bib-0002] Axelsson, J. , Rippe, A. , Oberg, C. M. , & Rippe, B. (2012). Rapid, dynamic changes in glomerular permeability to macromolecules during systemic angiotensin II (ANG II) infusion in rats. American Journal of Physiology. Renal Physiology, 303(6), F790–F799. 10.1152/ajprenal.00153.2012 22718887

[phy271147-bib-0003] Banas, M. C. , Banas, B. , Hudkins, K. L. , Wietecha, T. A. , Iyoda, M. , Bock, E. , Hauser, P. , Pippin, J. W. , Shankland, S. J. , Smith, K. D. , Stoelcker, B. , Liu, G. , Gröne, H. J. , Krämer, B. K. , & Alpers, C. E. (2008). TLR4 links podocytes with the innate immune system to mediate glomerular injury. Journal of the American Society of Nephrology, 19(4), 704–713. 10.1681/ASN.2007040395 PMC2390962 18256364

[phy271147-bib-0004] Bellinghieri, G. , Savica, V. , & Santoro, D. (2008). Renal alterations during exercise. Journal of Renal Nutrition, 18(1), 158–164. 10.1053/j.jrn.2007.10.031 18089464

[phy271147-bib-0005] Cho, H. , & Kim, J. H. (2017). Prevalence of microalbuminuria and its associated cardiometabolic risk factors in Korean youth: Data from the Korea National Health and nutrition examination survey. PLoS One, 12(6), e0178716. 10.1371/journal.pone.0178716 PMC5456094 28575100

[phy271147-bib-0006] Cho, R. L. , Yang, C. C. , Lee, I. T. , Lin, C. C. , Chi, P. L. , Hsiao, L. D. , & Yang, C. M. (2016). Lipopolysaccharide induces ICAM‐1 expression via a c‐Src/NADPH oxidase/ROS‐dependent NF‐κB pathway in human pulmonary alveolar epithelial cells. American Journal of Physiology. Lung Cellular and Molecular Physiology, 310(7), L639–L657. 10.1152/ajplung.00109.2014 26747779

[phy271147-bib-0007] Collino, F. , Bussolati, B. , Gerbaudo, E. , Marozio, L. , Pelissetto, S. , Benedetto, C. , & Camussi, G. (2008). Preeclamptic sera induce nephrin shedding from podocytes through endothelin‐1 release by endothelial glomerular cells. American Journal of Physiology. Renal Physiology, 294(5), F1185–F1194. 10.1152/ajprenal.00442.2007 18287402

[phy271147-bib-0008] Dickson, K. , & Lehmann, C. (2019). Inflammatory response to different toxins in experimental sepsis models. International Journal of Molecular Sciences, 20(18), 4341. 10.3390/ijms20184341 PMC6770119 31491842

[phy271147-bib-0009] Doublier, S. , Ruotsalainen, V. , Salvidio, G. , Lupia, E. , Biancone, L. , Conaldi, P. G. , Reponen, P. , Tryggvason, K. , & Camussi, G. (2001). Nephrin redistribution on podocytes is a potential mechanism for proteinuria in patients with primary acquired nephrotic syndrome. American Journal of Pathology, 158(5), 1723–1731. 10.1016/S0002-9440(10)64128-4 PMC1891937 11337370

[phy271147-bib-0010] Dryer, S. E. , Roshanravan, H. , & Kim, E. Y. (2019). TRPC channels: Regulation, dysregulation and contributions to chronic kidney disease. Biochimica et Biophysica Acta, Molecular Basis of Disease, 1865(6), 1041–1066. 10.1016/j.bbadis.2019.04.001 30953689

[phy271147-bib-0011] Forrester, S. J. , Booz, G. W. , Sigmund, C. D. , Coffman, T. M. , Kawai, T. , Rizzo, V. , Scalia, R. , & Eguchi, S. (2018). Angiotensin II signal transduction: An update on mechanisms of physiology and pathophysiology. Physiological Reviews, 98(3), 1627–1738. 10.1152/physrev.00038.2017 PMC6335102 29873596

[phy271147-bib-0012] Haraldsson, B. , Nyström, J. , & Deen, W. M. (2008). Properties of the glomerular barrier and mechanisms of proteinuria. Physiological Reviews, 88(2), 451–487. 10.1152/physrev.00055.2006 18391170

[phy271147-bib-0013] Hayashida, K. , Bartlett, A. H. , Chen, Y. , & Park, P. W. (2010). Molecular and cellular mechanisms of ectodomain shedding. Anatomical Record (Hoboken, N.J.: 2007), 293(6), 925–937. 10.1002/ar.20757 PMC4621804 20503387

[phy271147-bib-0014] Hu, X. , Zhou, W. , Wu, S. , Wang, R. , Luan, Z. , Geng, X. , Xu, N. , Zhang, Z. , Ruan, Z. , Wang, Z. , Li, F. , Yu, C. , & Ren, H. (2022). Tacrolimus alleviates LPS‐induced AKI by inhibiting TLR4/MyD88/NF‐κB signalling in mice. Journal of Cellular and Molecular Medicine, 26(2), 507–514. 10.1111/jcmm.17108 PMC8743665 34889045

[phy271147-bib-0015] Huh, W. , Kim, D. J. , Kim, M. K. , Kim, Y. G. , Oh, H. Y. , Ruotsalainen, V. , & Tryggvason, K. (2002). Expression of nephrin in acquired human glomerular disease. Nephrology, Dialysis, Transplantation, 17(3), 478–484. 10.1093/ndt/17.3.478 11865096

[phy271147-bib-0016] Hunt, J. L. , Pollak, M. R. , & Denker, B. M. (2005). Cultured podocytes establish a size‐selective barrier regulated by specific signaling pathways and demonstrate synchronized barrier assembly in a calcium switch model of junction formation. Journal of the American Society of Nephrology, 16(6), 1593–1602. 10.1681/ASN.2004080679 15843471

[phy271147-bib-0017] Keogh, B. , & Parker, A. E. (2011). Toll‐like receptors as targets for immune disorders. Trends in Pharmacological Sciences, 32(7), 435–442. 10.1016/j.tips.2011.03.008 21529972

[phy271147-bib-0018] Kim, E. Y. , Choi, K. J. , & Dryer, S. E. (2008). Nephrin binds to the COOH terminus of a large‐conductance Ca^2+^‐activated K^+^ channel isoform and regulates its expression on the cell surface. American Journal of Physiology. Renal Physiology, 295(1), F235–F246. 10.1152/ajprenal.00140.2008 PMC2494500 18480178

[phy271147-bib-0019] Kim, E. Y. , & Dryer, S. E. (2025). TRPC6 effects on albumin permeation, nephrin shedding, and apoptosis in podocytes: Role of calcineurin and metalloproteases. Physiological Reports, 13(21), e70614. 10.14814/phy2.70614 PMC12573276 41165237

[phy271147-bib-0020] Königshausen, E. , Zierhut, U. M. , Ruetze, M. , Potthoff, S. A. , Stegbauer, J. , Woznowski, M. , Quack, I. , Rump, L. C. , & Sellin, L. (2016). Angiotensin II increases glomerular permeability by β‐arrestin mediated nephrin endocytosis. Scientific Reports, 6, 39513. 10.1038/srep39513 PMC5177899 28004760

[phy271147-bib-0021] Königshausen, E. , Zierhut, U. M. , Ruetze, M. , Rump, L. C. , & Sellin, L. (2024). A molecular mechanism for angiotensin II receptor blocker‐mediated slit membrane protection: Angiotensin II increases nephrin endocytosis via AT1‐receptor‐dependent ERK 1/2 activation. FASEB Journal, 38(17), e70018. 10.1096/fj.202400369R 39212304

[phy271147-bib-0022] Kriz, W. , & Lemley, K. V. (2017). Mechanical challenges to the glomerular filtration barrier: Adaptations and pathway to sclerosis. Pediatric Nephrology, 32(3), 405–417. 10.1007/s00467-016-3358-9 27008645

[phy271147-bib-0023] Kriz, W. , Shirato, I. , Nagata, M. , LeHir, M. , & Lemley, K. V. (2013). The podocyte's response to stress: The enigma of foot process effacement. American Journal of Physiology. Renal Physiology, 304(4), F333–F347. 10.1152/ajprenal.00478.2012 23235479

[phy271147-bib-0024] Legat, A. , Thomas, S. , Hermand, P. , Van Mechelen, M. , Goldman, M. , & De Wit, D. (2010). CD14‐independent responses induced by a synthetic lipid a mimetic. European Journal of Immunology, 40(3), 797–802. 10.1002/eji.200939992 20039305

[phy271147-bib-0025] Li, S. , Liu, Y. , He, Y. , Rong, W. , Zhang, M. , Li, L. , Liu, Z. , & Zen, K. (2020). Podocytes present antigen to activate specific T cell immune responses in inflammatory renal disease. The Journal of Pathology, 252(2), 165–177. 10.1002/path.5508 32686090

[phy271147-bib-0026] Li, Z. , Tan, D. , Lin, J. , Zhang, T. , Liu, B. , Zhao, B. , Liang, D. , Li, L. , Wei, X. , Lv, Z. , Wang, R. , & Hu, M. (2025). Podocyte TLR4 deletion alleviates diabetic kidney disease through prohibiting PKCδ/SHP‐1‐dependent ER stress and relieving podocyte damage and inflammation. Journal of Advanced Research, 82(25), 845–862. 10.1016/j.jare.2025.07.013 PMC13001067 40659086

[phy271147-bib-0027] Lorenzen, J. , Shah, R. , Biser, A. , Staicu, S. A. , Niranjan, T. , Garcia, A. M. , Gruenwald, A. , Thomas, D. B. , Shatat, I. F. , Supe, K. , Woroniecki, R. P. , & Susztak, K. (2008). The role of osteopontin in the development of albuminuria. Journal of the American Society of Nephrology, 19(5), 884–890. 10.1681/ASN.20070404861 PMC2386721 18443355

[phy271147-bib-0028] Luo, R. , Yao, Y. , Chen, Z. , & Sun, X. (2025). An examination of the LPS‐TLR4 immune response through the analysis of molecular structures and protein‐protein interactions. Cell Communication and Signaling, 23(1), 142. 10.1186/s12964-025-02149-4 PMC11921546 40102851

[phy271147-bib-0029] Ma, J. , Chadban, S. J. , Zhao, C. Y. , Chen, X. , Kwan, T. , Panchapakesan, U. , Pollock, C. A. , & Wu, H. (2014). TLR4 activation promotes podocyte injury and interstitial fibrosis in diabetic nephropathy. PLoS One, 9(5), e97985. 10.1371/journal.pone.0097985 PMC4026484 24842252

[phy271147-bib-0030] Myette, R. L. , Trentin‐Sonoda, M. , Landry, C. , Holterman, C. E. , Lin, T. , Burger, D. , & Kennedy, C. R. J. (2025). Damage‐associated molecular patterns and pattern recognition receptors in the podocyte. Journal of the American Society of Nephrology, 36(1), 136–143. 10.1681/ASN.0000000531 PMC11706563 39331471

[phy271147-bib-0031] Pavenstädt, H. , Kriz, W. , & Kretzler, M. (2003). Cell biology of the glomerular podocyte. Physiological Reviews, 83(1), 253–307. 10.1152/physrev.00020.2002 12506131

[phy271147-bib-0032] Ravanelli, N. , Barry, H. , Schlader, Z. J. , & Gagnon, D. (2021). Impact of passive heat acclimation on markers of kidney function during heat stress. Experimental Physiology, 106(1), 269–281. 10.1113/EP088637 32495481

[phy271147-bib-0033] Reiser, J. , Kriz, W. , Kretzler, M. , & Mundel, P. (2000). The glomerular slit diaphragm is a modified adherens junction. Journal of the American Society of Nephrology, 11(1), 1–8. 10.1681/ASN.V1111 10616834

[phy271147-bib-0034] Reiser, J. , von Gersdorff, G. , Loos, M. , Oh, J. , Asanuma, K. , Giardino, L. , Rastaldi, M. P. , Calvaresi, N. , Watanabe, H. , Schwarz, K. , Faul, C. , Kretzler, M. , Davidson, A. , Sugimoto, H. , Kalluri, R. , Sharpe, A. H. , Kreidberg, J. A. , & Mundel, P. (2004). Induction of B7‐1 in podocytes is associated with nephrotic syndrome. Journal of Clinical Investigation, 113(10), 1390–1397. 10.1172/JCI20402 PMC406528 15146236

[phy271147-bib-0035] Rossol, M. , Heine, H. , Meusch, U. , Quandt, D. , Klein, C. , Sweet, M. J. , & Hauschildt, S. (2011). LPS‐induced cytokine production in human monocytes and macrophages. Critical Reviews in Immunology, 31(5), 379–446. 10.1615/critrevimmunol.v31.i5.20 22142165

[phy271147-bib-0036] Ruotsalainen, V. , Ljungberg, P. , Wartiovaara, J. , Lenkkeri, U. , Kestilä, M. , Jalanko, H. , Holmberg, C. , & Tryggvason, K. (1999). Nephrin is specifically located at the slit diaphragm of glomerular podocytes. Proceedings of the National Academy of Sciences of the United States of America, 96(14), 7962–7967. 10.1073/pnas.96.14.7962 PMC22170 10393930

[phy271147-bib-0037] Shankland, S. J. , Pippin, J. W. , Reiser, J. , & Mundel, P. (2007). Podocytes in culture: Past, present, and future. Kidney International, 72(1), 26–36. 10.1038/sj.ki.5002291 17457377

[phy271147-bib-0038] Shin, H. M. , Kim, M. H. , Kim, B. H. , Jung, S. H. , Kim, Y. S. , Park, H. J. , Hong, J. T. , Min, K. R. , & Kim, Y. (2004). Inhibitory action of novel aromatic diamine compound on lipopolysaccharide‐induced nuclear translocation of NF‐kappaB without affecting IkappaB degradation. FEBS Letters, 571(1–3), 50–54. 10.1016/j.febslet.2004.06.056 15280016

[phy271147-bib-0039] Stöver, A. G. , Da Silva Correia, J. , Evans, J. T. , Cluff, C. W. , Elliott, M. W. , Jeffery, E. W. , Johnson, D. A. , Lacy, M. J. , Baldridge, J. R. , Probst, P. , Ulevitch, R. J. , Persing, D. H. , & Hershberg, R. M. (2004). Structure‐activity relationship of synthetic toll‐like receptor 4 agonists. Journal of Biological Chemistry, 279(6), 4440–4449. 10.1074/jbc.M310760200 14570885

[phy271147-bib-0040] Takashima, K. , Matsunaga, N. , Yoshimatsu, M. , Hazeki, K. , Kaisho, T. , Uekata, M. , Hazeki, O. , Akira, S. , Iizawa, Y. , & Ii, M. (2009). Analysis of binding site for the novel small‐molecule TLR4 signal transduction inhibitor TAK‐242 and its therapeutic effect on mouse sepsis model. British Journal of Pharmacology, 157(7), 1250–1262. 10.1111/j.1476-5381.2009.00297.x PMC2743844 19563534

[phy271147-bib-0041] Tan, R. J. , Li, Y. , Rush, B. M. , Cerqueira, D. M. , Zhou, D. , Fu, H. , Ho, J. , Beer Stolz, D. , & Liu, Y. (2019). Tubular injury triggers podocyte dysfunction by β‐catenin‐driven release of MMP‐7. JCI Insight, 4(24), e122399. 10.1172/jci.insight.122399 PMC6975262 31743113

[phy271147-bib-0042] Thorp, E. , Vaisar, T. , Subramanian, M. , Mautner, L. , Blobel, C. , & Tabas, I. (2011). Shedding of the Mer tyrosine kinase receptor is mediated by ADAM17 protein through a pathway involving reactive oxygen species, protein kinase Cδ, and p38 mitogen‐activated protein kinase (MAPK). Journal of Biological Chemistry, 286(38), 33335–33344. 10.1074/jbc.M111.263020 PMC3190938 21828049

[phy271147-bib-0043] Tosetti, F. , Alessio, M. , Poggi, A. , & Zocchi, M. R. (2021). ADAM10 site‐dependent biology: Keeping control of a pervasive protease. International Journal of Molecular Sciences, 22(9), 4969. 10.3390/ijms22094969 PMC8124674 34067041

[phy271147-bib-0044] Vehaskari, V. M. , & Rapola, J. (1982). Isolated proteinuria: Analysis of a school‐age population. The Journal of Pediatrics, 101(5), 661–668. 10.1016/s0022-3476(82)80287-4 7131137

