# Supplementary material for: TLR4 signaling cascades leading to increases in albumin permeability, nephrin ectodomain shedding, and apoptosis in MPC‐5 podocytes
Source: Physiol Rep. 2026 Oct 16;14(20):e71147. doi: 10.14814/phy2.71147 (PMC13673513; doi:10.14814/phy2.71147)
Supplement: Supplementary file 1 [file PHY2-14-e71147-s001.pdf]

Supplemental Data

TLR4 signaling cascades leading to increased albumin permeability, nephrin ectodomain shedding and apoptosis in podocytes

Eun Young Kim and Stuart E. Dryer

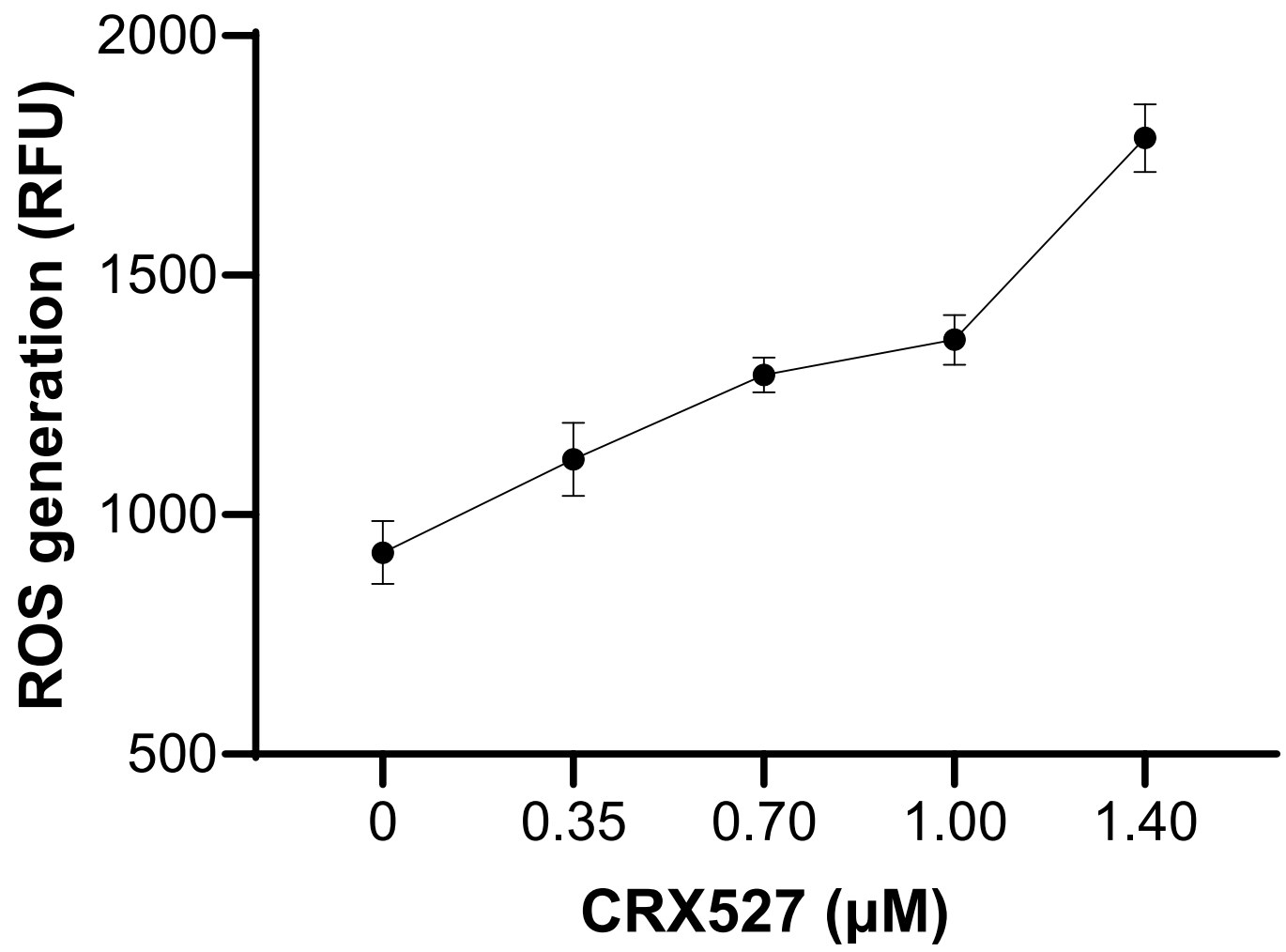

Supplemental Figure 1. Effects of CRX527 on ROS generation after 24 h. The experiments used in the body of the MS utilized 0.7 µM.

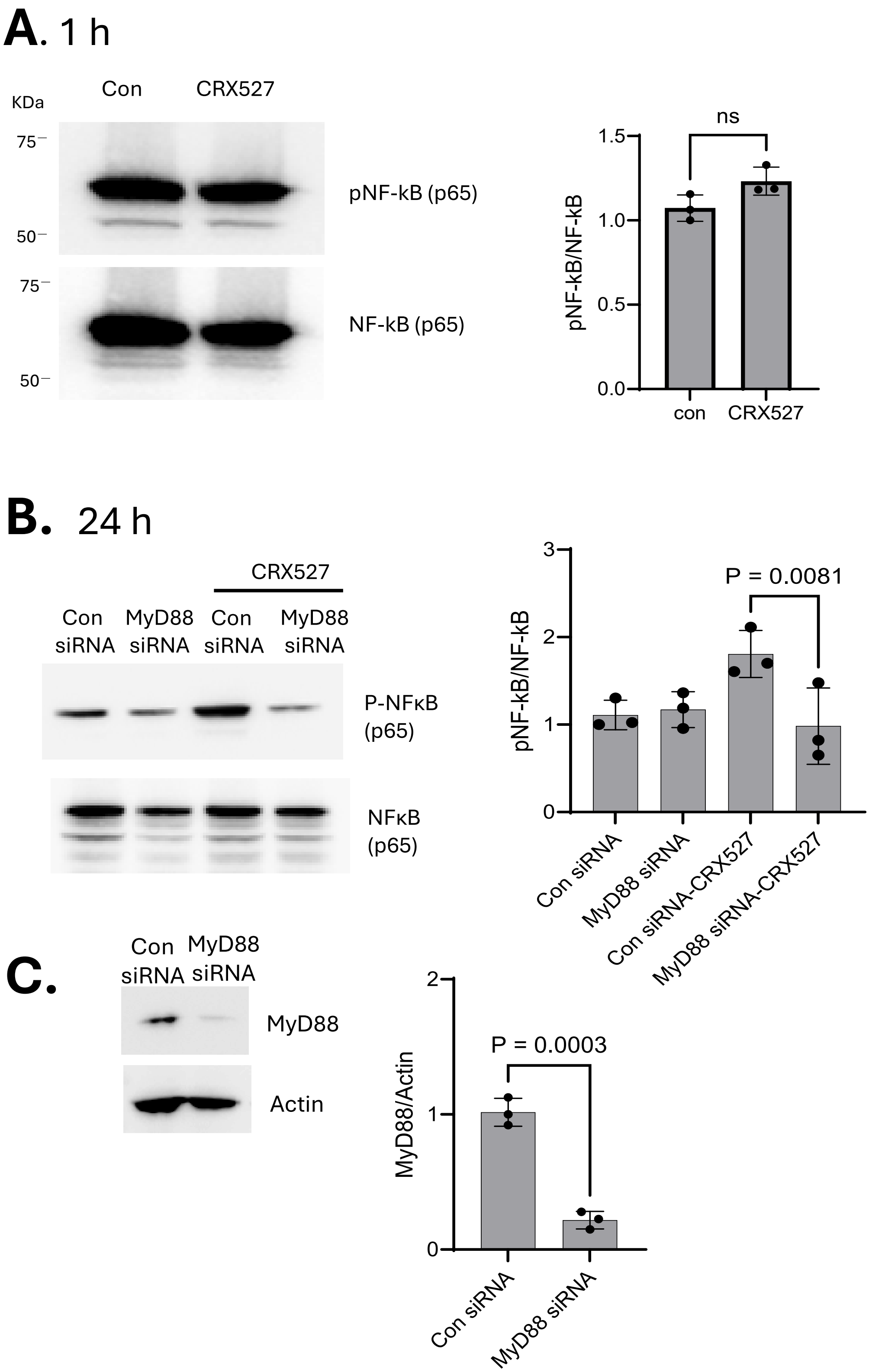

Supplemental Figure 2. MyD88 and NfKB activation at different time points (A, B) and at 24 h and after siRNA knockdown of MyD88 (C)

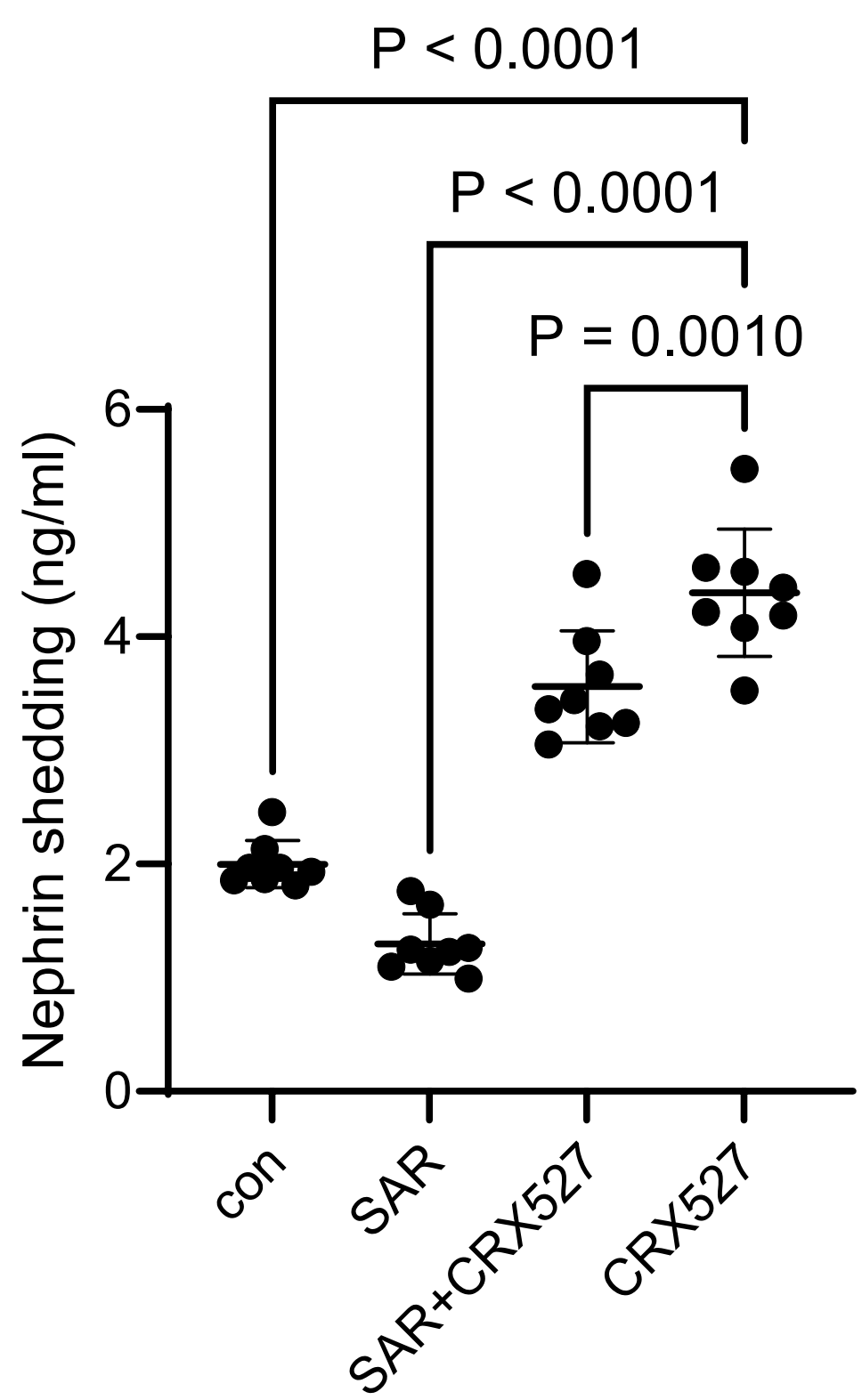

Supplemental Figure 3. Significant nephrin ectodomain shedding persists following blockade of TRPC6 channels
